# Study on Nanomaterials with Inhibitory Effects on the Growth of *Aspergillus niger*

**DOI:** 10.3390/polym15183820

**Published:** 2023-09-19

**Authors:** Ziqi Qin, Yiyuan Peng, Yiting Pu, Tao Liu, Kun Qian, Huan Tang

**Affiliations:** 1 College of Plant Protection, Southwest University, Chongqing 400715, China; qinziqi2021@163.com (Z.Q.); yiyuan10192023@163.com (Y.P.); puyiting2021@outlook.com (Y.P.); 13290042332@163.com (T.L.); qiankun1982@163.com (K.Q.); 2 Key Scientific Research Institution of Pest and Mold Control of Heritage Collection, China Three Gorges Museum, State Administration of Cultural Heritage, Chongqing 400015, China

**Keywords:** *Aspergillus niger*, copper nanorods, chitosan@copper nanorods

## Abstract

In this paper, the inhibitory effect of various nanomaterials on the growth of *Aspergillus niger* was studied. Among them, copper nanorods had the most obvious inhibitory effect on the growth of *Aspergillus niger*. The phase of copper nanorods was modified by chitosan, and its inhibitory effect on the expansion of *Aspergillus niger* was measured. 1. Preparation of copper nanorods and chitosan@copper nanorods: Copper nanorods with a diameter of about 300–350 nm and a length of about 100–800 nm were prepared by the liquid-phase reduction method. The chitosan solution was prepared by using the characteristics of chitosan dissolved in dilute acid to prepare chitosan@copper nanorods and modify the phase of copper nanorods. 2. Determination of the inhibitory effect of various copper nanomaterials on the growth of *Aspergillus niger*, including Cuprous Oxide nanoparticles, copper nanorods, nano copper oxide, and copper hydroxide, which have certain inhibitory effects on the growth of *Aspergillus niger*. Among them, copper nanorods have a better effect. On this basis, chitosan@copper nanorods are obtained by modifying the phase of copper nanorods with chitosan. The measured antibacterial effect is that the EC_50_ value is 344 mg/L.

## 1. Introduction

*Aspergillus niger* is a common species of *Aspergillus* fungi in the subgenus Trichosporae, order Trichosporae, and family Trichosporae. It is widely distributed in China and the world and is most commonly found in food, crop products, and soil. *Aspergillus niger* (*Aspergillus niger*) mainly causes post-harvest disease (post-harvested disease). High temperatures and humid environments are prone to cause it, especially in the sugar content of fruit and grain crops, which causes more serious damage. In grapes, for example, mould contamination can cause losses of up to 30–40% of the total yield of grapes and other bulk fruit and vegetables [1]. Where *Aspergillus niger* is a common pathogen on grapes, the fruit and tissues are slightly brownish after damage, gradually becoming soft and rotten, and the fruit stalks and fruit surface often grow a thick layer of mould, the conidia and conidia of the pathogen. The mould layer is white and thin when it starts to appear and becomes greenish-black and thicker when it forms in large numbers. In severe cases, most of the grape grains on the whole bunch are partially discoloured, or even yellowish white, and eventually the affected fruits lose water and crumple. *Aspergillus niger* is one of the dominant moulds in stored rice, and its strong mould-causing ability and fungal toxin production potential pose a serious threat to the safety of rice. In recent years, innovative use of tea tree essential oil [2], antagonistic bacteria such as Bacillus cereus [3], anthocyanin [4], etc. to inhibit the occurrence of *Aspergillus niger* has been reported [5], but silver materials are relatively expensive and the cost of control is high, so experiments have been conducted to explore the inhibitory effect of more nanomaterials on the growth of *Aspergillus niger* on this basis in order to explore other materials with good inhibitory effects on the growth of *Aspergillus niger*. The experiments were carried out to explore other materials with good inhibitory effects on the growth of *Aspergillus niger*.

Nanomaterials are materials with at least one-dimensional size between 1 and 100 nm, with tiny size and new structure between crystalline and non-crystalline [6], under the effect of their own quantum size effect, small size effect, dielectric limit effect, surface effect, and other effects [7], giving them new properties that are different from other bulk materials. Once introduced, they were widely studied by scholars from all walks of life. In recent years, nanomaterials have been studied, from nanoparticles and nanocomposites to nano-assembled systems, which have become a new hotspot and have been widely used in food, electronics, chemicals, medicine, textiles, and bioengineering, with great potential applications [8,9]. It has been shown that nanosilver-based materials have considerable inhibitory effects on the growth of moulds, which offers the possibility to continue to explore the inhibitory effects of other nanomaterials on the growth of *Aspergillus niger* in this paper. Since the discovery of carbon nanotubes by Lijima et al. in 1991 [10], the structure and properties of one-dimensional nanomaterials have attracted a great deal of interest from scholars in related fields. In the past three decades, a large number of 1D nanomaterials have been synthesised involving dozens of metals such as copper, iron, aluminium, magnesium, zinc, silver, nickel, manganese, cobalt, ruthenium, and platinum, which are used in a variety of fields such as electronics manufacturing, electrochemistry, and bioengineering. The following section will focus on copper nanorods, a 1D copper nanomaterial.

There are many methods for the preparation of copper nanorods, including vapour phase deposition and hydrothermal synthesis. Among them, hydrothermal synthesis is an excellent experimental method that is simple and easy to operate and requires low experimental conditions. Large-scale production of copper nanorods offers the possibility of preparing copper nanorods, usually under high temperature and pressure conditions, in a liquid-phase system in a closed vessel. Qingchun Chen prepared copper nanorods [11] with a width of 100–500 nm and a diameter of a micrometer by using CuSO_4_-5H_2_O and NaOH as the main materials and a hexa-aliphatic alcohol as the reducing agent and adjusting n(OH^−^):n(Cu^2+^) = 4 at 180 °C for 20 h under high temperature and high pressure conditions. In this experiment, the temperature, the amount of alcohol, and n(OH^−^):n(Cu^2+^) all had an important influence on the results, and the high temperature and the polyol were favourable to the production of copper nanorods, which gave us important ideas for our experiments. Due to the hydrophobic nature of copper nanorods, their poor dispersion in water and their tendency to agglomerate and settle limit their application to a certain extent. Therefore, the surface modification of copper nanorods by chitosan.

The chemical name of chitosan is polyglucosamine (1-4)-2-amino-b-d glucose [12]. It is generally insoluble in water, soluble in organic solvents, and can be dissolved in dilute acids as the amino group can be protonated in acidic media. Numerous studies have shown that chitosan has an inactivating effect on many bacteria, fungi, and yeasts [13], with broad-spectrum inhibition and high inactivation rates for Gram-positive and Gram-negative bacteria and low cytotoxicity to mammals [14]. Due to its non-toxic, adsorptive, degradable, and bioactive characteristics, chitosan has been widely used in food, pharmaceutical, environmental, and agricultural fields [15]. The preparation of carvacrol-chitosan nanoparticles and their antibacterial activity were investigated by Chengyu Xing et al. [16], who demonstrated that chitosan nanosized with a smaller particle size and higher specific surface energy could improve the biological activity of chitosan nanoparticles. The use of chitosan nanoparticles embedded with antibacterial materials can improve the stability of antibacterial substances, control the release rate, shield the high concentration of odour and achieve a slow-release, long-lasting antibacterial effect, which can be applied to a variety of nanomaterials.

In this experiment, copper nanorods were formulated by liquid-phase synthesis. Based on the characteristics of hydrophobicity and poor water dispersion of copper nanorods, chitosan was used to modify their surface, i.e., chitosan@copper nanorods, which greatly enhanced their water dispersion performance and improved their bacterial inhibition ability to a certain extent. In addition, the inhibition effect of various metal nanomaterials on the growth of *Aspergillus niger* was measured, from which nanomaterials with a better inhibition effect on the growth of *Aspergillus niger* were selected, and the reasons for the difference in inhibition were briefly analysed to provide some reference for the research and application of metal nanomaterials in agricultural fungicides.

## 2. Materials and Methods

### 2.1. Main Reagents

Ethylenediamine, sodium hydroxide (flake), copper nitrate trihydrate, chitosan (Sinopharm, Beijing, China), ice acetic acid, and anhydrous ethanol were acquired from Chengdu Kolon Chemicals Co. (Chengdu, China) Magnesium hydroxide nanoparticles, copper oxide nanoparticles, copper nanoparticles, and copper nanorods were all prepared in our laboratory.

### 2.2. Preparation Methods

#### 2.2.1. Preparation of Copper Nanorods (CuNRs)

Copper nanorods were prepared under alkaline conditions using copper sulphate trihydrate as the copper source, hydrazine hydrate as the reducing agent, and ethylenediamine as the complexing agent, following the method of Liu et al. [17] with slight modifications. Twenty mL of 15 M NaOH were measured at room temperature at 25 °C in a 50 mL conical flask with a stopper added in advance with a magnetic stirrer. Next, 2.0 mL of 100 mM CuSO_4_-3H_2_O were added to the conical flask, placed on a magnetic stirrer, and stirred well to obtain a clear blue solution. Next, 300 µL of ethylenediamine and 22 µL of hydrazine hydrate were added in rapid succession while stirring and stirring stopped when the solution changed colour from blue to dark blue and gradually to blue-violet. The conical flask was transferred to an oven at 85 °C, and the reaction was carried out for 1 h. The solution was observed to be red in colour, with a fluffy, pore-like red-brown substance emerging from the surface and a little crumbly substance at the bottom. After cooling to room temperature, the solution was transferred to a centrifuge tube, centrifuged at 10,000 rpm for 5 min, washed three times with anhydrous ethanol, freeze-dried, and stored at 25 °C at room temperature.

#### 2.2.2. Preparation of Chitosan@Copper Nanorods (CS@CuNRs)

A 1% acetic acid solution was prepared by mass by taking 100 mL of acetic acid solution in a beaker and adding 1 g of chitosan (CS). The mouth of the cup was covered with sealing film, placed on a magnetic stirrer, and stirred vigorously until it dissolved completely. A transparent yellow solution (chitosan solution) was be obtained and set aside.

Twenty mg of copper nanorods were weighed and placed into a 100 mL beaker with a magnetic stirrer added in advance. Then, 20 mL of anhydrous ethanol solution was added, sealed with sealing film, put into a sonicator, sonicated evenly, and placed on a magnetic stirrer after the sealing film was removed. Then, 20 mL of the configured chitosan solution were added dropwise while stirring. Stirring continued for half an hour after the addition to make it wrap evenly. After half an hour, transfer to a centrifuge tube, centrifuge at 10,000 rpm for 5 min, wash once with pure water, freeze-dry, and store at 25 °C at room temperature.

### 2.3. Strain Preservation and Culture Methods

Preservation of *Aspergillus niger*: *Aspergillus niger* was inoculated in the centre of a potato glucose agar medium (PDA plate) incubated at 28 °C for 7 d with light. An appropriate amount of mycelium was picked from the plate, placed in a seed-keeping tube with 15% glycerol, and placed in a refrigerator at 4 °C for cold storage. For long-term storage, it is placed in a freezer at −80 °C for freezing.

Culture of *Aspergillus niger*: When using, an appropriate amount of *Aspergillus niger* mycelium is picked in the centre of the PDA plate, placed upside down in a constant temperature incubator at 28 °C, and incubated until the colony is just as long as the edge of the Petri dish before use.

### 2.4. Inhibition Rate of Growth of Aspergillus niger by Four Agents

The toxicity of magnesium hydroxide nanoparticles, copper oxide nanoparticles, copper nanoparticles, and copper nanorods was determined in vitro using the plate with drug method [18]. Magnesium hydroxide nanoparticles, copper oxide nanoparticles, and copper nanorods were dissolved in sterile water; copper nanorods and copper nanoparticles were treated with a cell crusher to homogenise them; magnesium hydroxide nanoparticles and copper oxide nanoparticles were sonicated with an ultrasonicator to homogenise them; and a 10,000 mg/L master batch was prepared (ready to use). The master mixes of Mg(OH)_2_ nanoparticles, CuO nanoparticles, and Cu nanorods were diluted 10 times with sterile water (the total amount of liquid added to the medium was 1 mL). 9 mL of PDA medium was added to a 50 mL conical flask, sterilised, and cooled to 70 °C. 1 mL of the drug solution was added to it and mixed well to make the PDA medium with the drug. Three plates were set up for each nanomaterial and different drug concentrations, with an additional set of blank controls (1 mL of sterile water in PDA medium), and each concentration treatment group was repeated three times.

Inoculation of *Aspergillus niger* by the single-spore isolation method (refer to Buy New Red’s method with slight modifications) [19]. Ten mL/dish of sterilised PDA plates are prepared in advance. The inoculum ring was dipped in 75% alcohol, and overcooked (note: external flame of alcohol lamp), repeated 3 times, and left to cool to room temperature. The plate of *Aspergillus niger* strain was placed in the light at 28 °C for about 7 d, with colonies just to the edge of the Petri dish, and colonies covered with black spores were dipped in the mould layer and scribed on the plate to separate the conidia with sufficient spacing. The plates were incubated at 28 °C for 7 d. When inoculated with single spores, the plates were removed and set aside. The surface of the scalpel was disinfected by dipping it in 75% alcohol and burning it on the outer flame of an alcohol lamp, which was repeated 3 times, and then cooled to room temperature. Under a 40–45X dissecting microscope, the tip of the scalpel was used to pick the germinated single conidia from the scribing plate and transfer them to the medicated plate. Equation (1) is used to calculate the growth inhibition rate of black moulds.

(1) $$X=\frac{d_{1}-d_{2}}{d_{1}}\times 100\%\;$$

In the above equation,

X—growth inhibition rate;

*d*_1_—control colony diameter;

*d*_2_—colony diameter of the treatment group.

The results of the inhibition of the growth of *Aspergillus niger* by the above four nanomaterials were measured at a concentration of 1000 mg/L as follows.

(data results processed using SPSS 26).

## 3. Analysis of the Results

### 3.1. Preparation and Characterisation of Copper Nanomaterials

The prepared copper nanomaterials were characterised using X-ray diffraction (XRD) and infrared spectroscopy (FT-IR) at zeta potential, and the morphological characteristics of the copper nanomaterials were analysed using transmission electron microscopy (TEM). The above process was commissioned by an external institutional academic research council, and the results obtained are fed back as follows.

**Figure 1:**
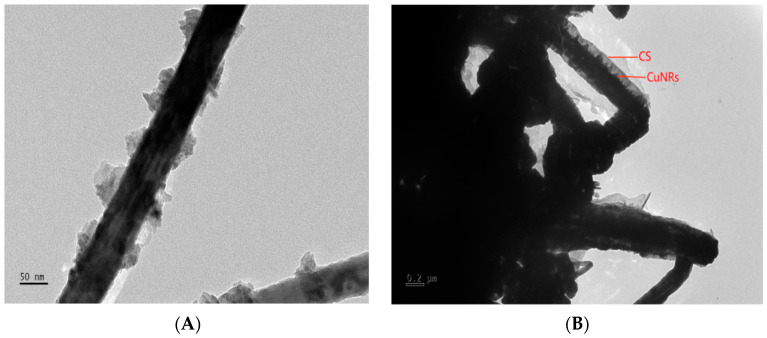
(**A**,**B**) TEM image of CuNRs and CS@CuNRs.

Figure 1A,B shows the TEM images of Cu nanorods and Cu nanorods modified with chitosan, from which it can be seen that CuNRs are thicker and shorter with a rough and concave surface, with a diameter of about 100–350 nm and a length of 100–800 nm, while CS@CuNRs have a smoother surface, with a diameter of about 200–400 nm and a length of 400–2000 nm. The CS is wrapped around the CuNRs as shown in the figure, making the CS@CuNRs longer and thicker than the CuNRs.

**Figure 2:**
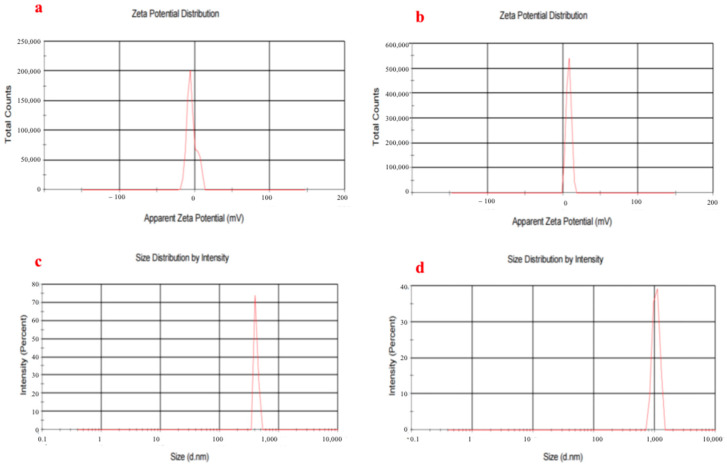
(**a**,**b**) Zeta (potential) image of CuNRs and CS@CuNRs; (**c**,**d**) the particle size profiles of Cu nanorods and Cu nanorods modified with chitosan.

Figure 2a,b shows the potential profiles of copper nanorods and copper nanorods with surface-modified chitosan. Diffractograms obtained the potential of CuNRs as −3.87 mA and CS@CuNRs as +7.88 mA. It is known that chitosan contains a large amount of amino groups that are positively charged [18]. The changes in potentials of CS@CuNRs are due to their surfaces being covered with a layer of positively charged chitosan, which is preliminary proof that CS@ CuNRs were successfully prepared.

Figure 2c,d shows the particle size profiles of Cu nanorods and Cu nanorods modified with chitosan, which shows that the particle size of CuNRs is 412.4 nm and the particle size of CS@CuNRs is 1056 nm.

**Figure 3:**
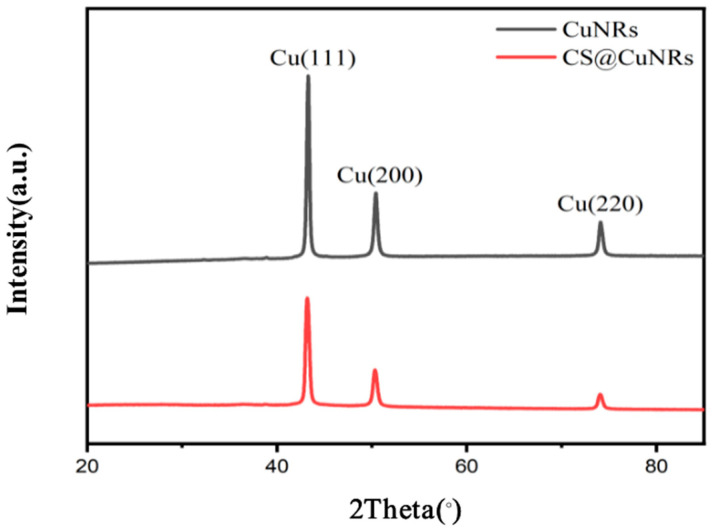
XRD image of CuNRs and CS@CuNRs.

Figure 3 shows the XRD patterns of the prepared Cu nanorods and Cu nanorods with chitosan surface modification. The patterns indicate that the CuNRs and CS@CuNRs have three identical main diffraction peaks on their surfaces, with no other impurity peaks and a good agreement with the (111), (200), and (220) crystallographic reflection peaks of Cu. This demonstrates that the prepared Cu nanorods and Cu nanorods with surface modification of chitosan are free of impurities, and the zero-valent Cu is not oxidised.

**Figure 4:**
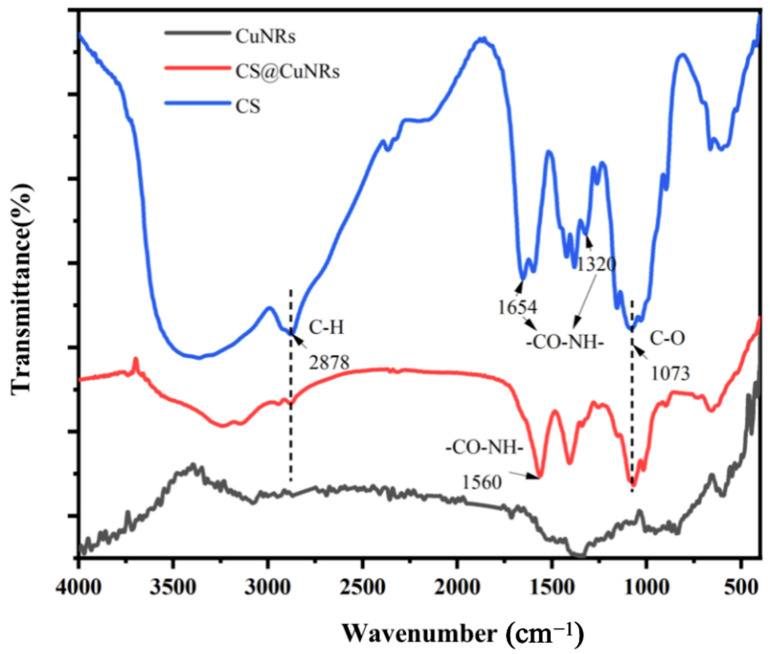
FT-IR image of CuNRs and CS@CuNRs.

Figure 4 shows the infrared (IR) spectra of Cu nanorods, Cu nanorods with surface modified chitosan, and chitosan, from which it can be seen that there is no obvious peak in the curve of CuNRs, which proves that there is no obvious oxide layer on the surface of the prepared Cu rods, and it can be enough to see that they have a C-H bond, a -CO-NN- bond and a C-O bond in CS. The CS@CuNRs also has -CO-NN- and C-O bonds, which can prove that the CS is indeed successfully wrapped around the CuNRs.

Homogeneous copper nanorods with diameters of 100–350 nm and lengths of 100–800 nm were prepared by liquid phase reduction and modified with chitosan. The modified copper nanorods, i.e., chitosan@copper nanorods, had diameters of approximately 200–400 nm and lengths of 400–2000 nm. The prepared copper nanomaterials were characterised by X-ray diffraction (XRD) and infrared spectroscopy (FT-IR) at zeta potential, and the morphological characteristics of the copper nanomaterials were analysed by transmission electron microscopy (TEM), both of which demonstrate that chitosan was successfully encapsulated on the surface of the copper nanorods.

#### Determination of the Growth Inhibition of *Aspergillus niger* Mycelium by Chitosan@Copper Nanorods

From the above results, it can be seen that when the concentration of the agents was 1000 ppm, the inhibition effect of the four agents on *Aspergillus niger* was significantly different, among which the inhibition effect of copper nanorods on the growth of *Aspergillus niger* mycelium was more obvious. Therefore, a smaller concentration gradient was set on this basis, and chitosan was used to modify the copper nanorods to make them more stable in order to further investigate the inhibition effect of copper nanorods on the growth of mycelium.

Chitosan@Cu nanorods were subjected to an in vitro indoor virulence assay using the drug-carrying plate culture method. Chitosan@Cu nanorods were dissolved in sterile water, treated with a cell crusher to homogenise them, and configured into a 10,000 mg/L master mix for reserve (ready-to-use). The master mix was diluted 10 times the mass concentration gradient in Table 1 with sterile water (the total amount of drug added to the medium was 1 mL). Each concentration treatment group was replicated four times and the remaining treatments were as in Section 2.4.

**Figure 5:**
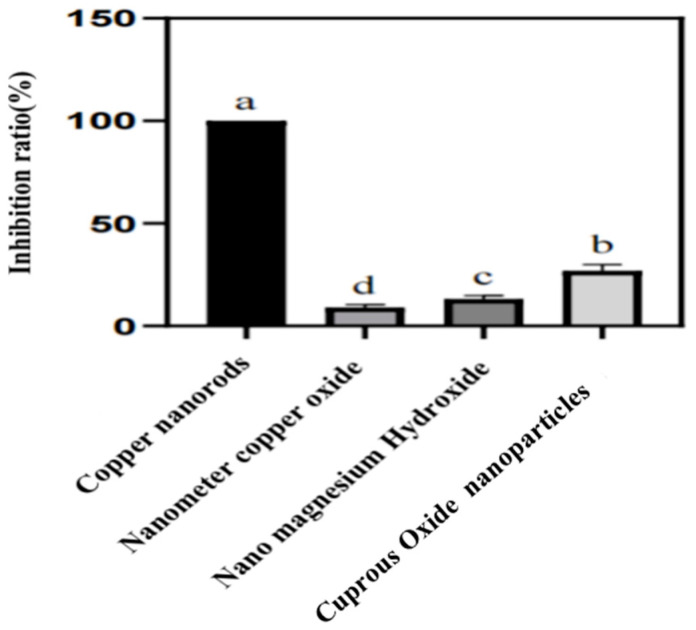
Bacteriostatic rate of four nanomaterials. (a, b, c, d represents the comparison results of differences between different groups).

As can be seen from Figure 5, the colony diameter decreased significantly with increasing concentrations of chitosan@copper nanorods, and the conidial growth was also inhibited. The colony diameters at different concentrations are shown in Table 2. The PDA culture was yellowish in colour and the plates were slightly greenish due to the oxidation of copper in the drug. The colour of the plates became darker as the concentration of the drug increased.

**Figure 6:**
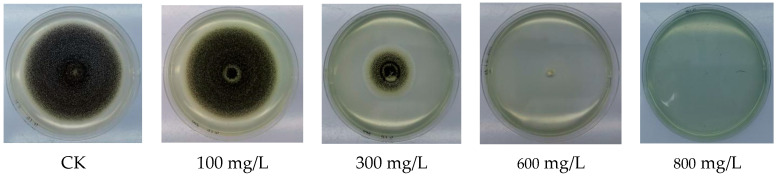
Inhibitory effect of CuNRs on the growth of *Aspergillus niger*.

The inoculation of *Aspergillus niger* by the single spore isolation method was carried out as described in Section 2.4 The growth of colonies at different concentrations of the solution was observed when the water control reached exactly the edge of the Petri dish, as shown in Figure 6.

**Figure 7:**
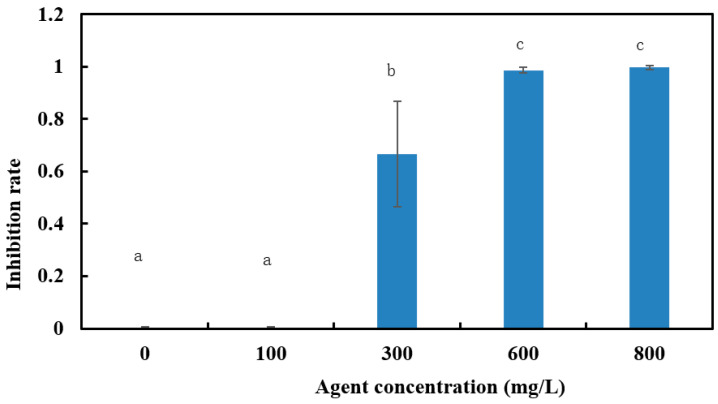
Histogram of the inhibitory effect of CS@CuNRs on the growth of *Aspergillus niger*. (a, b, c represents the comparison results of differences between different groups).

From Figure 7, it can be seen that the inhibition effect of CS@CuNRs was not obvious at low concentrations and increased significantly with increasing concentrations. The virulence regression curves of chitosan@CuNRs on the growth of *Aspergillus niger* were obtained according to Table 2, as shown in Table 3.

In this part, we firstly discussed the inhibitory effect of several nanomaterials, including copper nanorods, copper nanoparticles, copper oxide nanoparticles, and magnesium hydroxide nanoparticles, on the growth of *Aspergillus niger*. The inhibition rate of the four materials was measured by the strip plate method with all four solutions at 1000 mg/L, in which the inhibition effect of copper nanorods was significantly better than the other materials. Based on this, CS@CuNRs were obtained by surface modification of copper nanorods through chitosan to enhance their bacterial inhibition effect and stability in water. The regression equation of the virulence of CS@CuNRs on the growth of *Aspergillus niger* was further measured by using the liquid culture method with drugs. The virulence regression equation for CuNRs against *Aspergillus niger* was 362 mg/L for EC_50_ and 862 mg/L for EC_90_. The virulence regression equation for chitosan@CuNRs was y = 0.0014 + 0.0186. The virulence regression equation for chitosan@copper nanorods was 344 mg/L for EC_50_ and 630 mg/L for EC_90_. It is evident that the inhibition effect of copper nanorods was enhanced by the surface modification of chitosan. However, this test did not involve the in vivo inhibition test, and the inhibition mechanism of copper nanorods is not known, which needs to be further investigated.

## 4. Conclusions

In this paper, a copper nanorod with a diameter of approximately 300–350 nm and a length of 100–800 nm was prepared using a liquid-phase reduction method in a strongly alkaline solution system with copper nitrate trihydrate as the copper source, ethylenediamine as the complexing agent, and hydrazine hydrate as the reducing agent in a hydrothermal system at 80 °C. The surface modification of the copper nanorod using chitosan can reduce the surface oxidation rate of the copper nanorod, increase their water solubility, and improve their antibacterial effect. The inhibition of the growth of isolated *Aspergillus niger* by various nanomaterials, including copper-based nanomaterials, copper nanorods, copper oxide nanoparticles, copper nanoparticles, and magnesium-based nanomaterials, magnesium hydroxide nanoparticles, was tested using the plate banding and single spore inoculation methods. The results show that copper nanorods significantly inhibit the growth of *Aspergillus niger* mycelium. The surface-modified chitosan copper nanorods were more effective in inhibiting the growth of *Aspergillus niger* than the surface-unmodified copper nanorods, but this paper only compares the size of the inhibition concentration of the two; the mechanism of the difference in their inhibition effect is not yet clear, and further research is needed. Secondly, only the inhibition effect of copper nanorods on the growth of isolated *Aspergillus niger* before and after surface modification was measured; on the one hand, no in vivo inhibition test was conducted, and on the other hand, the safety of the two materials was not assessed. A difficult problem is that due to the certain bactericidal properties of CuNO_3_, if not washed thoroughly, the residual Cu^+^ will cause certain errors in the experimental results. Although we have undergone repeated washing, we may still be unable to avoid trace amounts of Cu^+^ residue. How to minimise the residual amount of Cu^+^ is also a problem that needs to be optimised in the future. Finally, field use is the ultimate goal of pesticide screening, as mould rot caused by *Aspergillus niger* is the dominant post-harvest disease, so the application scenario is mostly for post-harvest crop protection, but copper nanorods are dark reddish-brown in colour and direct application will affect the fruit appearance to a certain extent. Overall, although agents with good inhibitory effects on the growth of *Aspergillus niger* have now been screened, further research is needed in many aspects, from the inhibition mechanism to the in vivo inhibition effect to the final use, but I believe that the future is promising.

## Figures and Tables

**Table 1:** Mass concentration of test nanomaterials and agent for mycelial growth test (active ingredients.

Pharmaceuticals	Masterbatch mg/L	Mass Concentration mg/L			
		1	2	3	4
Chitosan@Copper nanorods	10,000	100	300	600	800

**Table 2:** Bacteriostatic effects of different liquid concentrations (fresh weight).

Concentration	Colony Diameter (cm)				Inhibition Rate (Mean)
	Repeat 1	Repeat 2	Repeat 3	Repeat 4	
CK	7.50	7.50	7.55	7.45	0 ± 0.54%
100	7.45	7.80	7.65	7.50	−1.33 ± 2.11%
300	0.40	2.40	3.75	3.45	66.67 ± 20.20%
600	0.00	0.10	0.20	0.10	98.67 ± 1.09%
800	0.00	0.00	0.00	0.10	99.67 ± 0.67%

**Table 3:** Toxicity of chitosan@copper nanorods to the growth of *Aspergillus niger*.

Materials/Fungicides Name	Toxicity Regression Equation	R^2^	EC_50_/(mg/L)	EC_90_/(mg/L)
Chitosan@Copper nanorods	y = 0.0014 + 0.0186	0.8842	344	630

## Data Availability

Not applicable.
